# Analyzing the Therapeutic Mechanism of Mongolian Medicine *Zhonglun*-5 in Rheumatoid Arthritis Using a Bagging Algorithm with Serum Metabonomics

**DOI:** 10.1155/2022/5997562

**Published:** 2022-12-07

**Authors:** Xiye Wang, Mingyang Jiang, Dan Li, Liang Xu

**Affiliations:** ^1^College of Chemistry and Materials Science, Inner Mongolia Minzu University, Tongliao 028000, China; ^2^Inner Mongolia Key Laboratory of Chemistry for Natural Products Chemistry and Synthesis for Functional Molecules, Tongliao 028000, China; ^3^College of Computer Science and Technology, Inner Mongolia Minzu University, Tongliao 028000, China

## Abstract

Rheumatoid arthritis (RA) is a complex autoimmune disorder. *Zhonglun-*5 (ZL), a traditional Mongolian medicine, exhibits an excellent clinical effect on RA; however, its molecular mechanism remains unclear. In this study, rat serum metabolomic analysis was performed to identify potential biomarkers for RA and investigate its treatment mechanism. A Dionex Ultimate 3000 ultrahigh-performance liquid chromatography system coupled with a Q-Exactive Focus Orbitrap mass spectrometer was used for metabonomics analysis. Bootstrap aggregation (bagging) classification algorithm was applied to process data from control (CG), model (MG), and treatment administration groups. The classification accuracy was 100.00% (6/6) in the decision tree model and 83.33% (5/6) in the *K*-nearest neighbor (KNN) model, accompanied by 18 training samples and 6 testing samples. Using volcanic map analysis, 24 biomarkers were identified between CG and MG, including those related to glycosphingolipid biosynthesis, arachidonic acid, fatty acids, amino acids, bile acids, vitamins, and sphingolipids. A set diagram of the heatmap and drug-biomarker network of potential biomarkers was constructed. After ZL administration, the levels of these biomarkers returned to normal, indicating that ZL had a therapeutic effect in rats with RA. This study established a solid theoretical foundation to promote further research on the clinical applicability of ZL.

## 1. Introduction

The etiology of rheumatoid arthritis (RA), an autoimmune disease, remains unknown. RA is mainly characterized by inflammatory synovitis and extra-articular organ involvement. In the worst case, the joints become deformed, leading to the loss of function. RA pathogenesis is related to heredity, infection, and sex hormone levels [1, 2]. Inflammatory cell infiltration, pannus formation, and cartilage destruction occur in the diseased joint, which can cause great pain in patients. At present, the main therapeutic drugs for RA are allopathic medicines; many of these drugs have been reported to induce toxicity and side effects that reduce patients' quality of life. Patient immunity may be significantly impaired after taking certain medicines, increasing the risk of bacterial infection and cancer. Therefore, its clinical application is limited.

Traditional Mongolian medicine includes a complete theoretical system for treating disease by aiming to comprehensively regulate body function. *Zhonglun*-5 (ZL), a traditional Mongolian medicine, is composed of *Sophora flavescens Ait* (SFA), *Gardenia jasminoides Ellis*, *Fructus toosendan*, *Terminalia chebularensis*, and *Lomatogonium rotatum*. As a traditional treatment for hot yellow water disease, swimming pain syndrome, gout, and other diseases, it has been reported to induce the effects of clearing heat, cooling blood, relaxing tendons, and relieving pain. Clinically, ZL has exhibited significantly beneficial effects on RA [3]. As the primary active component of ZL, SFA plays a vital role in inhibiting RA by regulating the Th1/Th2 cytokine response in RA via attenuated NF-*κ*B signaling, thereby inhibiting disease progression [4]. Oxymatrine is a monomeric alkaloid extracted from SFA that can regulate the imbalance between regulatory T cells and helper T17 cells in RA rats, yielding a good protective effect against certain diseases [5]. Although SFA itself has a strong therapeutic effect, ZL may exhibit a unique molecular mechanism against RA.

At present, metabonomics data are primarily classified using principal component analysis approaches, and a large amount of original data are often lost during data processing. If the cumulative contribution rate of the first several principal components is low, the model is unqualified, and the obtained classification accuracy is very low. The bootstrap aggregation (bagging) algorithm is a group learning algorithm used in the field of machine learning. As a representative algorithm in the data-mining field, bagging can be combined with other classification and regression algorithms to improve accuracy and stability and avoid overfitting [6]. Compared to traditional omics data classification methods, this algorithm does not lose any original data. In addition, the bagging algorithm is advantageous for solving omics data classification problems associated with small sample sizes, high dimensions, and sparseness [7]. For example, owing to the differences in functional brain tissues between individuals, improving the reproducibility of neuroimaging measurements is the main obstacle to the development of human neuroscience. Recent research results show that bagging can improve the reproducibility of functional zoning without long-term scanning. In calculating two large datasets, it was proven that bagging improves the repetition accuracy of cortical and subcortical functional zoning under a series of different parameter conditions compared to the standard clustering framework [8]. Furthermore, in research addressing novel Coronavirus-19 (COVID-19) diagnosis, Zhang et al. proposed a bagging dynamic deep learning network (B-DDLN) to diagnose COVID-19 using intelligent recognition of chest symptoms in X-ray images. The calculation results showed that the accuracy of B-DDLN was 98.8889%, and B-DDLN had the best diagnostic performance among the existing open image set diagnostic methods [9]. If the bagging algorithm is applied to the classification of metabonomics data, it can not only significantly improve the accuracy of metabonomics data classification but also be of great significance for expanding existing classification methods. After data processing, disease-related biomarkers were screened, and the regulatory effects of ZL on markers related to metabolic pathways were investigated. This study revealed the molecular mechanisms underlying the role of ZL in RA treatment.

## 2. Experimental

### 2.1. Chemicals and Reagents

ZL was purchased from the Affiliated Hospital of Inner Mongolia Minzu University (Tongliao, China). ZL powder was dissolved in 0.5% carboxymethyl cellulose sodium aqueous solution up to concentrations of 0.43 g/mL and stored at 4°C for animal experimentation. Complete Freund's adjuvant (CFA) was purchased from Sigma Chemical Co. (St. Louis, MO, USA). Methanol and formic acid were purchased from Thermo Fisher Technology Co. Ltd. (China). Superoxide dismutase (SOD), malondialdehyde (MDA), tumor necrosis factor-*α* (TNF-*α*), and interleukin-1*β* (IL-1*β*) were purchased from Shanghai Fantai Biotechnology Co. Ltd. (Shanghai, China).

### 2.2. Adjuvant-Induced Arthritis Model Design and Treatment

This study was approved by the Ethics Committee of the Affiliated Hospital of Inner Mongolia Minzu University (NMMZDX2020[K]0034). Adjuvant arthritis is an inflammatory reaction model mediated by cellular immunity. The antigen enters the body to sensitize T cells. When it contacts the antigen again, the sensitized T cells differentiate and proliferate, release various lymphokines, and directly kill target cells to cause inflammation. Multiple arthritis is the feature of this model, which is closer to clinical human rheumatoid arthritis. To develop an animal model of RA treatment with ZL, 24 male Wistar rats (200 ± 10 g) were purchased from Shenyang Aikesaisi Biotechnology Co., Ltd. (Shenyang, China). All animals were acclimatized to the laboratory for one week before the experiment. The rats were divided into three groups: control, model, and ZL administration groups (CG, MG, and ZL, respectively), with eight rats in each group. On day 1, MG and ZL rats were intradermally injected with 0.1 mL CFA in the right posterior toe, and CG rats were injected with 0.1 mL saline. After 7 days, MG and ZL rats were injected with 0.1 mL CFA again. On day 14, the rats in the ZL group were administered Zhonglun-5 at doses of 0.86 g/kg/day for 28 consecutive days, and on day 42, all rats were euthanized. Blood was collected from the hepatic portal vein and centrifuged at 3500 rpm for 10 min at 4°C. The supernatants were immediately frozen and stored at −20°C. The arthrodial cartilage was fixed in 10% formaldehyde for paraffin embedding [10].

### 2.3. Biochemical and Histological Analysis

The treated articular cartilage was stained with hematoxylin and eosin. The sections of articular cartilage were observed under a microscope. A Multiskan FC Microplate Reader (Fisher Scientific, USA) was used to measure changes in SOD, MDA, TNF-*α*, and IL-1*β* levels in different groups.

### 2.4. Serum Sample Preparation

The serum samples were thawed before analysis, and 100 *μ*L aliquots were added to 400 *μ*L acetonitrile, followed by vortexing for 30 s and centrifugation at 12000 rpm for 10 min at 4°C. The supernatant was subsequently filtered through a 0.22 *μ*m filter membrane.

### 2.5. UPLC-MS Conditions

A Thermo Dionex Ultimate 3000 UHPLC system coupled with a Q Exactive Focus Orbitrap mass spectrometer (Thermo, USA) was used for metabonomics analysis.

The Waters Acquity UPLC BEH C_18_ Column (1.7 *μ*m, 2.1 mm × 50 mm, Waters, UK) was maintained at 40°C with a flow rate of 0.3 mL·min^−1^ for separation. The mobile phases used were 0.1% formic acid in deionized water (A) and methanol (B). The gradient elution with B was performed according to the following schedule: 2% B for 0–0.5 min, 2%–95% B for 0.5–7 min, 95% B for 7-8 min, 95%–2% B for 8-8.1 min, and 2% B for 8.1–10 min. The sample injection volume was 10 *μ*L.

Optimized mass spectrum conditions were as follows: the flow rates of the sheath and auxiliary gas were 45 and 10 bar, respectively. The spray voltage was 3.5 kV, and the capillary and auxiliary gas heater temperatures were 320 and 350°C, respectively.

The MS data were collected in switching mode between positive and negative spectra. The mass inspection range was 100–1000 Da. The resolution of the full MS was 70000. In the dd-MS^2^ discovery mode, the resolution was 17500. The MS^2^ collision energy was 35 eV.

### 2.6. Data Analysis

Every day, eight pooled quality control samples were used to test the stability of the instrument. Peak detection, alignment, and normalization were performed using Compound Discoverer software (CD, version 2.0). MATLAB 2012 software was used to process the data based on the bagging algorithm. CD was used to analyze the volcanic map, while the Statistical Package for the Social Sciences (SPSS, version 20.0) was applied for an independent sample *t*-test. Potential biomarkers were screened according to the multiple content change (>2 times the content change) and *P* value (*P* < 0.05). Heml software was used to show intergroup changes in biomarkers [11]. The Human Metabolome Database was used to identify potential biomarkers. A set diagram of the heatmap and drug-biomarker network of potential biomarkers was constructed using Cytoscape 3.2.1.

### 2.7. Application of Bagging Algorithm for Classification

In this study, metabonomic data on ZL for the treatment of RA were classified. This dataset consists of three types of samples (CG, MG, and ZL administration groups, *n* = 8 for each group, 24 samples in total), and the dimension of each sample data was 2286.

The bagging algorithm was used to complete the resampling of metabonomics data; that is, *k* new metabonomics datasets are selected from the original metabonomics dataset through bootstrap sampling to train the classification model. These new metabonomic datasets can then be repeated. The trained multiple classifiers are used to classify metabonomics samples, and the classification results of all classifiers are counted by majority voting or the output-averaging method. The category with the highest result was the final label. This method can reduce the overfitting problem associated with a single classification model, improve the learning effect, and generate accurate predictions.

**Figure 1:**
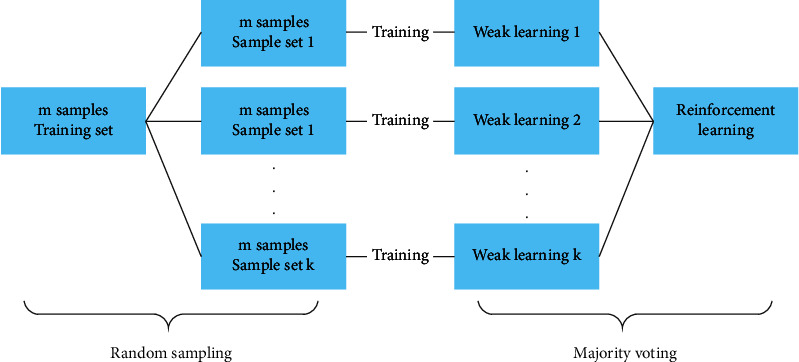
Bagging classification model.

To improve the difference in the model, when bagging trained the model for combination, data were randomly extracted from the training set. For the bagging algorithm, samples of the same number as the training set were randomly collected. Thus, the number of samples in the sampling set was the same as that in the training set, but the sample content was different. For example, if we randomly sample *k* times for the training set containing *n* samples, the *k* sample sets are different owing to randomness. This method usually considers homogeneous weak learners, learns them independently in parallel, and combines them according to a certain deterministic averaging process. The bagging classification model is illustrated in Figure 1.

If an ensemble model consisting of *k* classification models is built and we assume that the error of each model on each sample is *ϵ*_*i*_, the error obeys a multidimensional normal distribution with zero mean, *E*[*ϵ*_*i*_^2^]=*ν*variance, and *E*[*ϵ*_*i*_*ϵ*_*j*_]=*c* covariance. The average prediction error obtained from all ensemble classification models is $1/k\underset{i}{\sum }\epsilon^{i}$, and the mathematical expectation of the square error is as follows:

(1) $$\begin{array}{c} E\left[{\left(\frac{1}{k}\underset{i}{\sum }\epsilon^{i}\right)}^{2}\right]=\frac{1}{k^{2}}E\left[\underset{i}{\sum }\left(\epsilon_{i}^{2}+\underset{j\neq i}{\sum }\epsilon_{i}\epsilon_{j}\right)\right]=\frac{1}{k}v+\frac{k-1}{k}c\text{.} \end{array}$$

When the error is completely correlated (*c* = *v*), the mean square error is reduced to *V*; thus, the ensemble model has no effect. If the error is completely unrelated (*c* = 0), the expected value of the ensemble square error is only 1/*k* × *v*, which means that the expected value of the ensemble square error will decrease linearly with an increase in the ensemble size. In other words, the integration approach should perform at least as well as any of the other approaches and significantly better than any of the other single approaches if the errors of each individual model are independent.

In this experiment, *n* training samples were randomly selected from the original sample set, and *k* rounds were extracted to obtain *k* training sets that were independent of each other and accompanied by repeatable elements. For *N* training sets, *K* decision trees and *K*-nearest neighbor (KNN) models were trained, and the final classification results were obtained by majority voting. KNN is a supervised classification algorithm. If a sample has *K* most similar samples in the feature space, and most of these samples belong to a certain category, then the sample also belongs to this category [12, 13]. In each experiment, samples were assigned to the training and test sets. The number of randomly selected training samples *N* was the same as the number of training set samples, and the rest of the test set samples were used to verify the classification accuracy. The classification experiment was completed by selecting the proportion of data for different training and test sets.

## 3. Results and Discussion

### 3.1. Biochemistry and Histology

**Figure 2:**
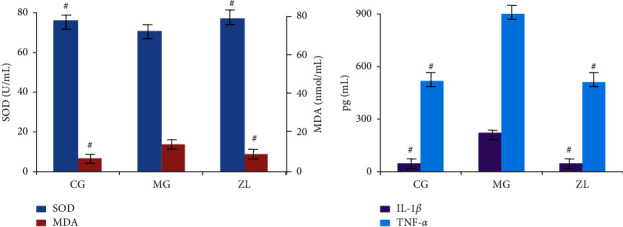
Indices of oxidant stress (a) and cytokine levels (b) in rat serum. Data are shown as mean ± standard deviation (*n* = 8). CG, control group; MG, model group; ZL, ZL administration group. ^#^*P* < 0.05 compared with MG.

The serum biochemical parameters are shown in Figure 2. As an antioxidant metalloenzyme in organisms, SOD promotes the conversion of superoxide anion radicals into oxygen and hydrogen peroxide, maintaining the balance between oxidation and antioxidation. Thus, it is crucial in many disease processes. MDA content is a parameter that reflects the potential antioxidant capacity in vivo. The increase in MDA content indicated that the degree of tissue peroxidation damage had increased. As shown in Figure 2(a), the SOD content decreased, and the MDA content increased in MG compared to CG, indicating that serious peroxidation occurred in RA rats. After ZL administration, the contents of SOD and MDA normalized, indicating that ZL exhibited antioxidant activity.

TNF-*α* and IL-1*β* are closely associated with RA development. They are produced by excessive secretion of matrix metalloproteinases and can cause severe damage to the joints. As shown in Figure 2(b), the levels of TNF-*α* and IL-1*β* increased significantly in the MG compared to those in the CG. After ZL administration, the levels of TNF-*α* and IL-1*β* decreased compared to those in MG, indicating that they all exhibit anti-inflammatory activity.

**Figure 3:**
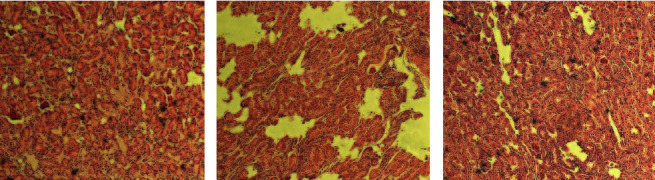
Histopathological change in rat arthrodial cartilage. Photomicrographs show representative cartilage sections stained with hematoxylin and eosin (100×). (a) Control group; (b) model group; (c) ZL administration group.

The histopathology of each group is shown in Figure 3. RA results in numerous panni (yellow stripes in Figure 3(b)) in the MG. Synovial hyperplasia, neovascularization, and leukocyte extravasation transform the normal acellular synovium into an invasive pannus. An imbalance in the microvascular structure leads to an insufficient synovial oxygen supply. With an increase in metabolic turnover of the dilated synovial pannus, a hypoxic microenvironment is formed in the synovium. Therefore, abnormal cell metabolism and mitochondrial dysfunction occur, which induces the production of RA [14]. After ZL administration (Figure 3(c)), the pannus was significantly reduced, indicating that ZL had a positive effect on RA.

### 3.2. Data Classification Results

**Figure 4:**
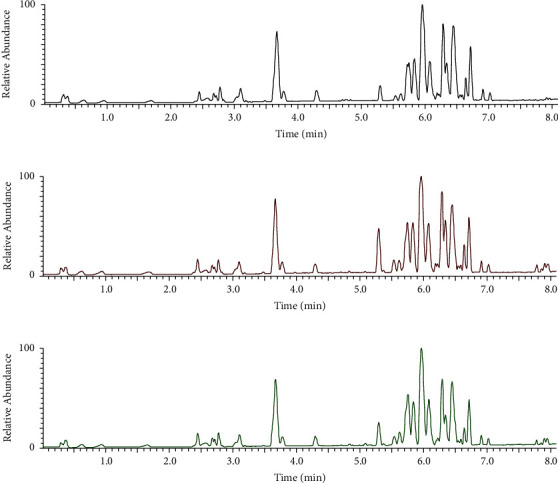
Serum total ion flow chromatograms of various groups. (a) Control group, (b) model group, and (c) ZL administration group in the switching mode between positive and negative spectra.

The serum total ion flow chromatograms of CG, MG, and ZL are shown in Figure 4. Small differences between the groups were observed using the bagging algorithm.

The data classification results are presented in Table 1. In general, the more training samples there are, the more reliable the model will be, and the higher the grouping accuracy. The results of the decision tree were better than those of KNN. The classification accuracy was 100.00% (6/6) in the decision tree model and 83.33% (5/6) in the KNN model (5/6 samples were correctly grouped), accompanied by 18 training samples and 6 testing samples. With 15 training samples and 9 testing samples, the classification accuracy was 88.89% (8/9) in the decision tree model and 77.78% (7/9) in the KNN model. The classification accuracy was 75.00% (9/12) in the decision tree model and 75.00% (9/12) in the KNN model, accompanied by 12 training samples and 12 testing samples. Finally, with 9 training samples and 15 testing samples, the classification accuracy was 73.33% (11/15) in the decision tree model and 66.67% (10/15) in the KNN model. The high classification accuracy laid a good foundation for screening biomarkers and inferring metabolic pathways.

### 3.3. Identification of Potential Biomarkers

**Figure 5:**
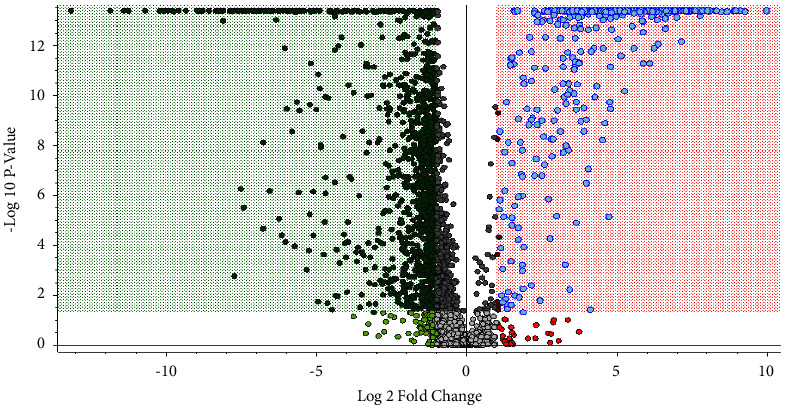
Volcano figure of serum metabolic profiling of CG and MG groups. Dots in green-shaded areas represent the biomarkers with decreased content (MG vs CG), and dots in red-shaded areas represent the biomarkers with increased content (MG vs CG).

**Figure 6:**
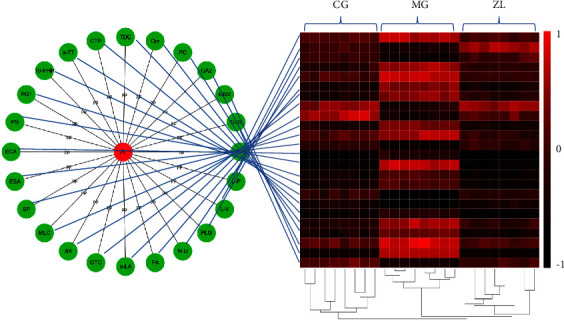
Set diagram of heatmap and drug-biomarker network of potential biomarkers regulated by ZL. (PG1, prostaglandin G1; 10-HHP, 10-hydroperoxy-H4-neuroprostane; GA2, ganglioside GA2 (d18 : 1/18 : 0); GD3, ganglioside GD3 (d18 : 1/23 : 0); GM2, ganglioside GM2 (d18 : 0/18 : 1 (11Z)); PA, palmitoleic acid; ESA, eicosapentaenoic acid; *α*-LA, *α*-linolenic acid; SA, stearic acid; OTC, octadecanamide; ECA, eicosadienoic acid; PLG, phenylacetyl glycine; L-V, L-valine; MLC, myristoyl glycine; N-U, N-undecanoylglycine; L-P, L-proline; TDC, tauroursodeoxycholic acid; GTS, gytosine; CTP, 13′-carboxy-*γ*-tocopherol; *α*-TT, *α*-tocotrienol; SP, sphinganine; PC, PC (22 : 5 (7Z, 10Z, 13Z, 16Z, 19Z)/16 : 0); Cer, cer (d18 : 0/16 : 0); PS, phytosphingosine).

A volcano model was used to detect potential biomarkers (Figure 5). Between CG and MG, 24 metabolites were screened that were involved in arachidonic acid metabolism, glycosphingolipid biosynthesis, fatty acid metabolism, amino acid metabolism, bile acid metabolism, vitamin metabolism, and sphingolipid metabolism. After ZL treatment, all metabolites returned to normal levels, indicating that ZL may have a therapeutic effect on RA by affecting various metabolic pathways. The set diagram of heatmap and drug-biomarker network of the potential biomarkers that can be regulated by ZL is shown in Figure 6. The biomarker information is summarized in Table 2.

### 3.4. Biological Relevance

Prostaglandins (PGs) comprise a type of lipid mediator produced by arachidonic acid metabolism and are abundant in bodily fluids. PGs combine with specific receptors and mediate multiple cellular activities, such as cell proliferation, differentiation, and apoptosis. In addition, PGs also participate in the pathological processes of inflammation, cancer, and various cardiovascular diseases [15]. PGs are lipid-signaling factors released during the early stages of RA. PGs maintain immune system inflammation by regulating the differentiation and maturation of immune cells and cytokine production. PGs are conducive to leukocyte infiltration, synovial hyperplasia, and angiogenesis in synovitis and participate in cartilage degradation and bone resorption. PGs are important mediators of joint pain regulation and can protect joints during the late stage of RA inflammation [16, 17]. PGs are lipid mediators produced by the enzymatic metabolism of arachidonic acid, an eicosanounsaturated fatty acid. NF-*κ*B is the main switch of proinflammatory genes, which can activate arachidonic acid pathway enzymes and lead to inflammation [18]. After ZL administration, the levels of prostaglandin G1 and 10-hydroperoxy-H4- neuroprostane returned to normal levels, indicating that ZL had a regulatory effect on arachidonic acid metabolism.

Gangliosides are important glycosphingolipids that are rich in nerve endings and assist in transmitting nerve impulses. In patients with RA, ganglioside levels in the synovium are significantly decreased compared to healthy patients. In the RA mouse model, ganglioside deficiency exacerbated inflammatory arthritis. In addition, the destruction of gangliosides can induce T cell activation in vivo and promote excessive production of RA-related cytokines. These findings suggest that gangliosides play key roles in RA pathogenesis and progression [19]. After ZL administration, ganglioside content normalized.

Fatty acid metabolism is closely associated with RA [20]. As a Ω-3 fatty acid (Ω-3 FA), eicosapentaenoic acid (EPA) is mainly found in fish oil. EPA has been found to inhibit inflammation via several mechanisms, including reducing the expression of adhesion molecules and T cell response activity, inhibiting the production of prostaglandins and leukotrienes by arachidonic acid, and inhibiting the production of inflammatory cytokines. The anti-inflammatory mechanisms of Ω-3 FAs include changing the composition of phospholipid fatty acids in cell membranes, destroying lipid rafts, and inhibiting the activation of proinflammatory transcription factors, thereby reducing the expression of inflammatory genes [21]. *α*-Linolenic acid inhibits arachidonic acid metabolism, resulting in the inhibition of the production of proinflammatory n-6 eicosanoids and a decrease in vascular permeability [22]. After ZL administration, the content of various fatty acids normalized.

RA can lead to an imbalance between amino acid and bile acid metabolism. Amino acid metabolism is a key regulator of immune response and can provide new drug targets for safer and more effective RA treatment [23]. Glycine is a potential immunomodulator that prevents reactive arthritis by increasing the influx of chloride ions through glycine-gated chloride channels and slowing the release of cytokines by macrophages [24]. Bile acid is an important component of bile that plays an important role in fat metabolism. MCP-induced protein (MCPIP) is a new zinc finger protein that participates in inflammatory angiogenesis. Tauroursodeoxycholic acid can block endoplasmic reticulum (ER) stress and inhibit inflammatory angiogenesis induced by MCPIP [25]. After ZL administration, the levels of various amino acids and bile acids returned to normal levels, indicating that ZL can regulate amino acid and bile acid metabolism.

RA affects vitamin E metabolism as a hydrolytic product of vitamin E, and tocopherol can be further metabolized to either 13′-carboxy-*γ*-tocopherol or *α*-tocotrienol. As a fat-soluble antioxidant, tocopherol captures free radicals produced by lipid oxidation in cell membranes and exhibits antioxidant effects [26]. Oxygen free radicals are considered mediators of tissue damage in RA patients. When the *α*-tocopherol content in the blood is low, the oxygen free radical cannot be removed efficiently, and the risk of RA increases [27]. After ZL administration, 13′-carboxy-*γ*-tocopherol and *α*-tocotrienol levels normalized.

RA can also lead to phospholipid and sphingolipid metabolism disorders. Phosphatidylcholine (PC) is an oily substance present in animal tissues and egg yolk. PCs mainly include phosphoric acid, choline, fatty acids, glycerol, glycolipids, triglycerides, and phospholipids. It is an important component of cell membranes, alveolar surfactants, lipoproteins, and bile as well as a source of lipid messengers such as lysophosphatidylcholine, phosphatidic acid, diglyceride, lysophosphatidic acid, and arachidonic acid [28]. PC and its derived metabolites have shown anti-inflammatory activity under various stress conditions. Experimental studies reported that rats fed PC exhibited reduced arthritis-induced hypersensitivity, the frequency of leukocyte-endothelial interactions, and the range of functional capillary density. PC also improves tissue damage by reducing the expression of nitric oxide synthase [29]. Ceramides are sphingosine lipids composed of sphingosine long-chain bases and fatty acids. Ceramides can regulate cell differentiation, proliferation, apoptosis, aging, and other life activities [30]. The balance between cell proliferation and apoptosis is impaired, which leads to excessive growth of synovial cells in rheumatoid joints and aggravates the destruction of joints. Ceramides mediate multiple cellular functions as lipid messengers. After ceramide pretreatment, the cell cycle process of synovial cells was completely inhibited, and the symptoms of RA were relieved [31]. Phytosphingosine is a ceramide precursor. As a lipid component of the skin, it exerts a natural repairing effect on barrier function. Studies have shown that phytosphingosine derivatives can effectively inhibit inflammatory response [32]. After ZL administration, PC (22 : 5 (7Z, 10Z, 13Z, 16Z, 19Z)/16 : 0), Cer (d18 : 0/16 : 0), and phytosphingosine contents normalized, indicating that ZL can regulate phospholipid and sphingolipid metabolism.

## 4. Conclusions

In summary, ZL alleviated RA in a rat model. The bagging algorithm was applied to process omics data, and the classification result was found to be outstanding based on the decision tree and the KNN model. A total of 24 biomarkers related to RA were identified involving multiple metabolic pathways, such as those related to glycosphingolipid biosynthesis, arachidonic acid, fatty acids, amino acids, bile acids, vitamins, and sphingolipid metabolism. After ZL administration, the levels of these biomarkers returned to normal. In future studies, we will examine the effects of ZL and its components on metabolic pathways in an RA rat model to clarify the compatibility mechanism of ZL.

## Figures and Tables

**Table 1:** Results of metabonomics data classification.

No.	Sampling frequency (*k*)	Number of training samples (*n*)	Number of testing samples (*m*)	Classification accuracy (%)	
				Decision trees	KNN
1	10	18	6	100.00 (6/6)	83.33 (5/6)
2	10	15	9	88.89 (8/9)	77.78 (7/9)
3	10	12	12	75.00 (9/12)	75.00 (9/12)
4	10	9	15	73.33 (11/15)	66.67 (10/15)

**Table 2:** Potential biomarkers between the control group and model group.

*t*_R_ (min)	Measured mass	10^6^ mass deviation	Chemical formula	Compound	Metabolic pathway
4.195	370.2345	2.7	C_20_H_34_O_6_	Prostaglandin G1	Arachidonic acid metabolism
8.131	392.2191	2.0	C_22_H_32_O_6_	10-hydroperoxy-H4-neuroprostane	Arachidonic acid metabolism
8.619	1092.7293	0.8	C_56_H_104_N_2_O_18_	Ganglioside GA2 (d18 : 1/18 : 0)	Glycosphingolipid biosynthesis
4.445	1541.9183	0.1	C_75_H_135_N_3_O_29_	Ganglioside GD3 (d18 : 1/23 : 0)	Glycosphingolipid biosynthesis
3.936	1382.8229	4.1	C_68_H_122_N_2_O_26_	Ganglioside GM2 (d18 : 0/18 : 1 (11Z))	Glycosphingolipid biosynthesis
0.097	254.2237	3.5	C_16_H_30_O_2_	Palmitoleic acid	Fatty acid metabolism
7.648	302.2244	0.7	C_20_H_30_O_2_	Eicosapentaenoic acid	Fatty acid metabolism
4.827	278.2246	0	C_18_H_30_O_2_	*α*-Linolenic acid	Fatty acid metabolism
3.487	284.2707	2.8	C_18_H_36_O_2_	Stearic acid	Fatty acid metabolism
6.150	283.2873	0.7	C_18_H_37_NO	Octadecanamide	Fatty acid metabolism
9.832	308.2701	4.5	C_20_H_36_O_2_	Eicosadienoic acid	Fatty acid metabolism
9.705	193.0739	0	C_10_H_11_NO_3_	Phenylacetylglycine	Amino acid metabolism
7.561	117.0792	1.7	C_5_H_11_NO_2_	L-valine	Amino acid metabolism
4.974	285.2302	0.7	C_16_H_31_NO_3_	Myristoylglycine	Amino acid metabolism
4.149	243.1833	0.4	C_13_H_25_NO_3_	N-undecanoylglycine	Amino acid metabolism
9.801	115.0637	3.5	C_5_H_9_NO_2_	L-proline	Amino acid metabolism
6.923	499.2961	1.4	C_26_H_45_NO_6_S	Tauroursodeoxycholic acid	Bile acid metabolism
6.700	111.0435	1.8	C_3_H_5_N_3_O	Gytosine	Pyrimidine metabolism
0.480	446.3386	2.2	C_28_H_46_O_4_	13′-Carboxy-*γ*-tocopherol	Vitamin E metabolism
5.517	424.3330	2.6	C_29_H_44_O_2_	*α*-Tocotrienol	Vitamin E metabolism
4.396	301.2979	0.7	C_18_H_39_NO_2_	Sphinganine	Sphingolipid signaling metabolism
4.826	807.5771	0.8	C_46_H_82_NO_8_P	PC (22 : 5 (7Z, 10Z, 13Z, 16Z, 19Z)/16 : 0)	Phospholipid metabolism
4.397	539.5281	0.7	C_34_H_69_NO_3_	Cer (d18 : 0/16 : 0)	Sphingolipid metabolism
4.597	317.2926	1.3	C_18_H_39_NO_3_	Phytosphingosine	Sphingolipid metabolism

## Data Availability

The data used to support the findings of this study are available from the corresponding author upon request.
